# The Toll-like receptor ligand, CpG oligodeoxynucleotides, regulate proliferation and osteogenic differentiation of osteoblast

**DOI:** 10.1186/s13018-020-01844-x

**Published:** 2020-08-14

**Authors:** Wenwen Yu, Yi Zheng, Hongyan Li, Hongbing Lin, Zhen Chen, Yue Tian, Huishan Chen, Peipei Zhang, Xiaowei Xu, Yuqin Shen

**Affiliations:** 1 Department of Periodontics, School and hospital of Stomatology, Jilin University, 1500 Qinghua Road, Changchun, Jilin, 130021 China; 2 Department of Orthodontics, Tianjin Key Laboratory of Oral and Maxillofacial Function Reconstruction; Tianjin Stomatological Hospital; Hospital of Stomatology, Nankai University, 75 Dagu North Road, Tianjin, 300041 China

**Keywords:** Periodontitis, CpG ODN, Osteoblast, Proliferation, Differentiation

## Abstract

**Background:**

This study aimed to investigate the regulation of CpG oligodeoxynucleotides (ODNs) on proliferation and osteogenic differentiation of MC3T3 cells.

**Methods:**

The laser co-focusing and flow cytometry assay were employed to detect cell uptake of CpG ODN 2006. Twelve ODNs were sythesized, and their effects on proliferation and differentiation were detected by MTT and alkaline phosphatase (ALP) activity assay. Flow cytometry assay was used to examine the regulation of CpG ODN on cell cycle. Quantitative real-time PCR (qRT-PCR) and western blot were used to evaluate the regulation of CpG ODN on mRNA and protein expression of osteogenic differentiation genes.

**Results:**

The phosphorothioate CpG ODN 2006 could efficiently enter the MC3T3 cells in 1 h and locate in the cytoplasm. The MTT assay demonstrated CpG ODNs could promote MC3T3 cell proliferation and differentiation in the early stage, and gradually attenuated along with the increase of treating time, except for BW001 and FC001. qRT-PCR assay demonstrated that all the 12 CpG ODNs could promote the relative expression level of osteogenic differentiated genes, SP7 and OCN. In addition, western blot analysis suggested the CpG ODNs of BW001 and FC001 could increase the protein expression of P27^Kip1^ and Runx2 and decrease the protein expression of cyclin D1.

**Conclusion:**

The selected CpGODNs may be a potential gene therapy for bone regeneration of periodontitis.

## Background

Periodontitis is caused by bacterial infection and natural immune response, and the cardinal symptoms are inflammation and bone absorption. Toll-like receptors (TLRs) are pathogen pattern recognition receptors, which play important roles in immunity response [1]. TLR9 could activate nuclear factor (NF)-kappa B and mitogen-activated protein kinase (MAPK) by canonical MyD88-interleukin 1 receptor-associated protein kinase (IRAK)-tumor necrosis factor receptor-associated factor 6 (TRAF6) pathway to initiate anti-infection immune response [2]. Furthmore, the TLR9 also expresses on the suface of osteocytes to regulate bone metabolism [3]. Agents involved in lipopolysaccharide signal transduction pathways induced by TLR has been proposed to be potential strategy for treating inflammation disease, such as allergic diseases [4]. Due to the significance of TLR on inflammatory and bone formation, we hyperthesized that agents that could target TLR might also have potential for treating periodontitis.

Bacterial DNA is a kind of pathogen-associated molecular pattern that specially recognizes TLR, and its immunological basis is the unmethylated CpG motif [5]. Studies have shown that either CpG-based sequences of bacterial or synthetic CpG oligodeoxynucleotides (ODNs) could strongly regulate immune function, which can quickly trigger the body to resist infection [6]. CpG ODN is widely used in the research and treatment of immunology. The mouse model and clinical trials have confirmed the immuno-stimulating effects of synthetic CpG ODN [7] on various diseases, including cancer, allergic diseases, asthma and infectious diseases [8, 9]. However, the role of CpG ODN on bone metabolism is far from understanded.

The previous researches have reported the tight association between CpG ODN and osteocytes. CpG ODN can regulate the differentiation of osteoclasts by binding to TLR9 [3, 10], and this regulation could also be indirectly achieved by targeting TLR9 of osteoblasts’ surface [10]. Actived TLR9 on osteoblast’s surface initiates extracellular signal-regulated protein kinase and P38 to release tumor necrosis factor α, then enhancing the expression of macrophage colony-stimulating factor to regulate osteoclasts [11]. The CpG ODN yet plays a positive role in osteogenic differentiaion of bone mesenchymal stem cells [12] and pre-osteoblasts [13].

In the past dacades, serveral CpG ODNs have been designed and their roles have been investigated widely [14, 15]. However, there were few studies about the relation of CpG ODN on osteogenic differentiation in previous researches. Shen et al. and Feng et al. have reported the effects of multiple CpG ODNs on bone marrow mesenchymal stem cells and MG63 cells [12, 13]. Nevertheless, the molecular mechanism of these CpG ODNs are largely unknown. In this study, we prepared 12 ODNs and investigated their roles on proliferation and differentiation of osteoclasts. We aimed to provide some CpG ODNs that have potential to be applied for bone regeneration on the basis of their anti-inflammation ability in treating periodontitis.

## Materials and methods

### Cell culture

The MC3T3 cells (GNM15) were purchased from Cell bank of Chinese Academy of Sciences (Shanghai, China) and were cultured in high glucose-Dulbecco’s modified Eagle’s medium (DMEM) supplemented with 10% fetal bovine serum (FBS) and 100 U/mL penicillin and 100 mg/mL streptomycin (ThermoFisher, Carlsbad, CA, USA). Cells were cultured in humidified atmosphere at 37 °C and 5% CO_2_.

Thirteen ODNs, including 2006, FC003, SAT05f, SAT05d, MS19, BW001, FC001, FC002, BW006, YW002, YW001, FC004, and MT01 were sythesized in Takara (Dalian, China), and the sequence was listed in Table 1.

**Table 1:** Gene sequence of the 13 ODNs and the primer sequence for quantitative real-time PCR

Number	Name	Sequence
	2006	5′-TCGTCGTTTTGTCGTTTTGTCGTT-3′
1	FC003	5′-TCTCTCTCTCTCTCTCTCTCTCTC-3′
2	SAT05f	5′-CCTCCTCCTCCTCCTCCTCCTCCT-3′
3	SAT05d	5′-CTCTCTCTCTCTCTCTCTCTCTCT-3′
4	MS19	5′-AAAGAAAGAAAGAAAGAAAGAAAG-3′
5	BW001	5′-TCGTCGGGTGCGACGTCGCAGGGGGG-3′
6	FC001	5′-TCGGGGACGATCGTCGGGGGG-3′
7	FC002	5′-TCGTCGACGTCGTTCGTTCTC-3′
8	BW006	5′-TCGACGTTCGTCGTTCGTCGTTC-3′
9	YW002	5′-TCGCGAACGTTCGCCGCGTTCGAACGCGG-3′
10	YW001	5′-TCGCGACGTTCGCCCGACGTTCGGTA-3′
11	FC004	5′-TCGCGAACGTTCGCCCGATCGTCGGTA-3′
12	MT01	5′-ACCCCCTCTACCCCCTCTACCCCCTCT-3′
	β-actin	5′-CATCCGTAAAGACCTCTATGCCAAC-3′ 5′-ATGGAGCCACCGATCCACA-3′
	SP7	5′-AAGTTATGATGACGGGTCAGGTACA-3′ 5′-AGAAATCTACGAGCAAGGTCTCCAC-3′
	OCN	5′-ACCATCTTTCTGCTCACTCTGCT-3′ 5′-CCTTATTGCCCTCCTGCTTG-3′

The MC3T3 cells were divided into 14 groups, including cells treated with phosphate buffer saline (PBS, control group), cells treated with ODN 2006 (positive control), and cells treated with the 12 kinds of ODNs.

### Laser co-focusing detection

MC3T3 cells were seeded on the glass slides in a 6-well plate at a density of 2 × 10^4^ cells/well and incubated for 24 h. Then, the cells were treated with 2 μg/mL Cy5-ODN 2006 for 1 h, washed with PBS for three times and fixed by 4% paraformaldehyde for 10 min. After being washed with 0.1% Triton X-100, the actin-tracker green work fluid (Beyotime Biotech Inc., Jiangsu, China) was added to cover the cells for 30 min in dark. The nucleus were stained by DAPI (Beyotime Biotech Inc., Jiangsu, China) for additional 5 min. After washing by PBS, the glass slides were fixed by 50% glycerol and detected by laser co-focusing apparatus (FV1000, Olympus, Osaka, Japan).

### Flow cytometry (FCM) assay

After transfected with Cy5-ODN for 1 h, cells were collected in the dark, then washed and centrifuged for three times before being transferred into transparent glass tubes. The indices of flow cytometry under green light were set at 540 nm wavelengths. The ratio of the Cy5-positive cells/total cells was determined quantitatively.

MC3T3 cells after co-culturing with the 12 kinds of ODNs and ODN 2006 for 72 h were trypsinized, collected, centrifuged and washed by cold PBS, then fixed by cold 70% ethyl alcohol for 24 h under 4 °C. After that, the cells were centrifuged and washed again to discard 70% ethyl alcohol. Then, the cells were stained with 1 mL propidium iodide (PI)/TritonX-100 staining solution (7sea biotech, Shanghai, China) at 37 °C for 30 min. Finally, the samples were tested by FACS caliber (BD Biosciences, San Jose, CA USA) and the cell ratio of G1, S and G2 phase was examined by using FlowJo software (BD Biosciences).

### 4,5-Dimethylthiazol-2-yl)-2,5-diphenyltetrazolium (MTT) assay

The MC3T3 cells were seeded into a 96-well plate with 3 × 10^3^ per well. After 24 h, PBS, ODN 2006 and the 12 kinds of ODNs were respectively added at a concentration of 2 μg/mL. After co-culturing for 24, 48and 72 h, 5 mg/mL MTT (Sigma-Aldrich, St Louis, MO, USA) was added to each well and incubated for another 4 h. Then, the MTT solution was removed and 150 μL DMSO (Sigma-Aldrich, St Louis, MO, USA) was added to dissolve the formazan crystals for 10 min. Absorbance was examined at 492 nm by a GF-M3000 microplate reader (Shandong, China).

### Alkaline phosphatase (ALP) activity assay and staining

The MC3T3 cells in the 96-well plate were cultured in osteogenic differentiation medium (1 × 10^− 8^ M dexamethasone, 10 mM β-glycerophosphate, and 50 μg/mL l-ascorbic acid) (Sigma-Aldrich, St Louis, MO, USA), then lysed using 1% Triton X-100 after treating with ODNs and 2006 for 24, 48 and 72 h. The total ALP activity and protein concentration were evaluated by using an ALP assay kit (Jiancheng Bioengineering institute, Nanjing, Jiangsu, China) and a bicinchoninic acid (BCA) protein assay kit (Beyotime Biotech Inc., Jiangsu, China). The ALP levels were normalized to the total protein content.

MC3T3 cells were seeded in a 24-well plate and were cultured in a conditioned medium with ODNs and ODN 2006 for 7 days. Then, ALP staining was performed using BCIP/NBT ALP color development kit (Beyotime Biotech Inc., Jiangsu, China). The stained cells in each group were photographed, and cell staining was independently repeated at least three times.

### Quantitative real-time reverse transcription polymerase chain reaction (qRT-PCR)

Total RNA of MC3T3 cells were extracted by Trizol reagent (ThermoFisher), and reverse-transcribed in cDNA using PrimerScript® RT reagent kit (Takara). After that, qRT-PCR was performed on MxPro Mx3005P system (Agilent Technologies, Santa Clara, CA, USA) using SYBR Green Premix Ex Taq kit (Takara). The cycling conditions of mRNAs were as follows: 95 °C for 30 s, followed by 40 cycles of 95 °C for 5 s, 55 °C for 30 s, and 72 °C for 1 min. The primer sequence was listed in Table 1. The relative expression levels were calculated by 2^−ΔΔCt^ method with β-actin as internal control. The obtained values were averaged from triplicate measurements.

### Western blot analysis

Total protein of MC3T3 cells were harvested with RIPA lysis buffer and quantified with a BCA assay kit (Beyotime). Proteins were separated on 12% SDS-polyacrylamide gels and transferred to polyvinylidene fluoride membranes (Millipore, Billerica, MA, USA). These membranes were blocked with 5% bull serum albumin in Tris buffered saline with Tween 20 and incubated with the following primary antibodies at 4 °C overnight: cyclin D1 (1:500, A2708, ABclonal, Wuhan, China), SP7 (1:1000, ab94744, Abcam, Shanghai, China), OCN (1:650, A5786, ABclonal), Runx2 (1:500, A2851, ABclonal), P27^Kip1^ (1:500, A2692, ABclonal), and β-actin (1:100000, AC026, ABclonal). Horseradish peroxidase-conjugated anti-rabbit IgG (H + L) secondary antibodies were added (1:3000, AS014, ABclonal) and incubated at 25 °C for 1 h. The signals were detected using an ECL chemiluminescence kit (7Sea biotech, Shanghai, China) by Tanon 5200 (Tanon, Shanghai, China).

### Statistical analysis

Statistical analysis was conducted by SPSS version 20.0 (IBM). All experiments were performed at least three times. Results were presented as the mean ± standard deviation (SD). Differences among groups were compared by one-way analysis of variance with Bonferroni post hoc test. A two-tailed *P* value < 0.05 was considered statistically significant.

## Results

### CpG ODN could be internalized by MC3T3 cells

**Fig. 1:**
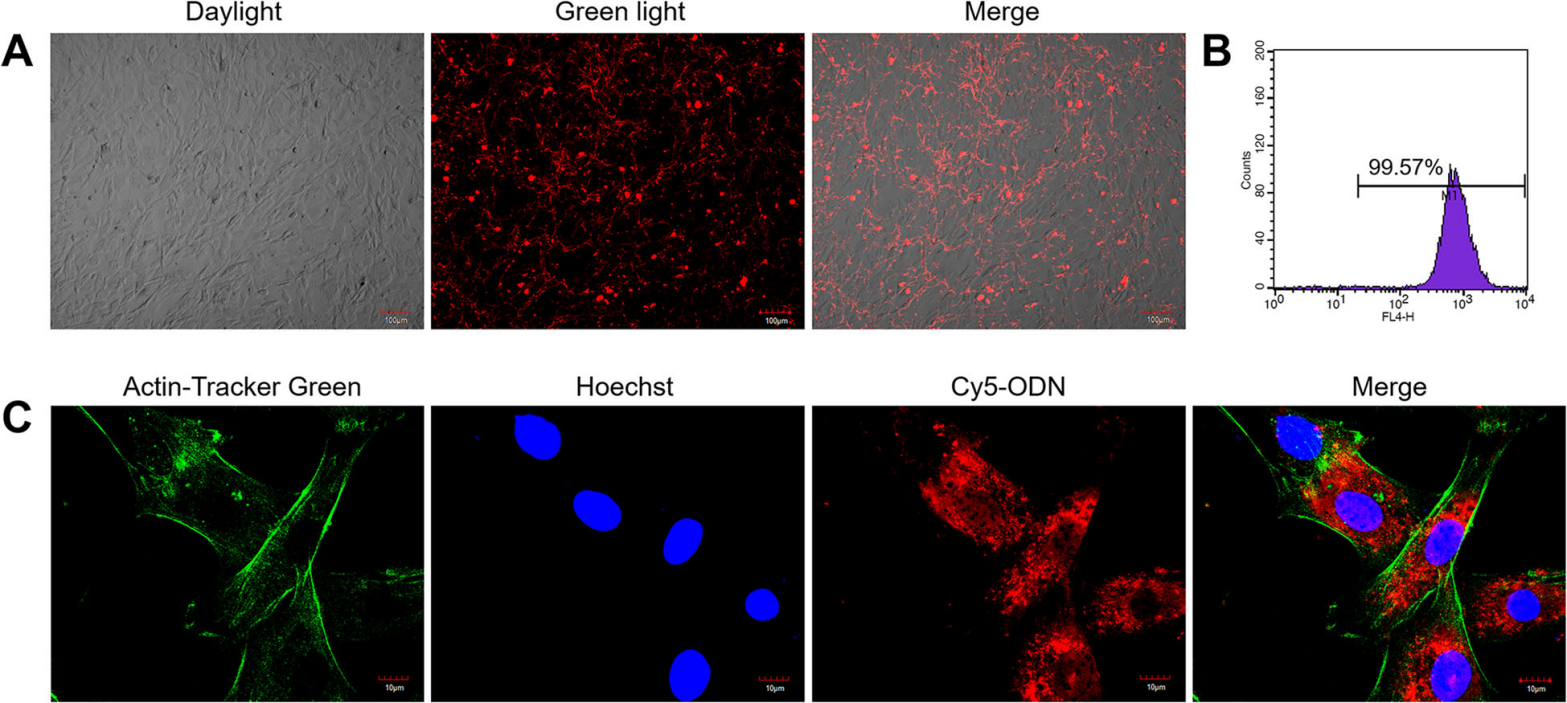
The uptake of CpG ODN 2006 by MC3T3 cells. **a** Microscopic images of Cy5-ODN 2006-positive MC3T3 cells under ordinary light, green light and merged of ordinary light and green light. Scale bar, 100 μm. *n* = 3. **b** Flow cytometry analysis of Cy5-ODN 2006-positive MC3T3 cells. *n* = 3. **c** Laser co-focusing detection of location of Cy5-ODN 2006 on MC3T3 cells. From left to right, the stained cells were detected by blue light, ultraviolet light, green light and merged of three. *n* = 3

In order to investigate whether ODNs could be uptaken by MC3T3 cells and to check its distribution in cells, we performed a laser confocal detection using ODN 2006. The results showed that the Cy5-labeled ODN 2006 could quickly penetrate into MC3T3 cells within 1 h, and the Cy5-ODN 2006 positive cells were estimated to be more than 90% according to fluorescence microscopy (Fig. 1a) and flow cytometry assay (Fig. 1b). The high magnification images displayed that ODN 2006 was mainly distributed in the cytoplasm and barely enter the nucleus (Fig. 1c). These results indicated that phosphorothioate ODN could enter cells quickly and efficiently and is mainly distributed in the cytoplasm.

### CpG ODNs influenced cell proliferation and cell cycle of MC3T3 cells

**Fig. 2:**
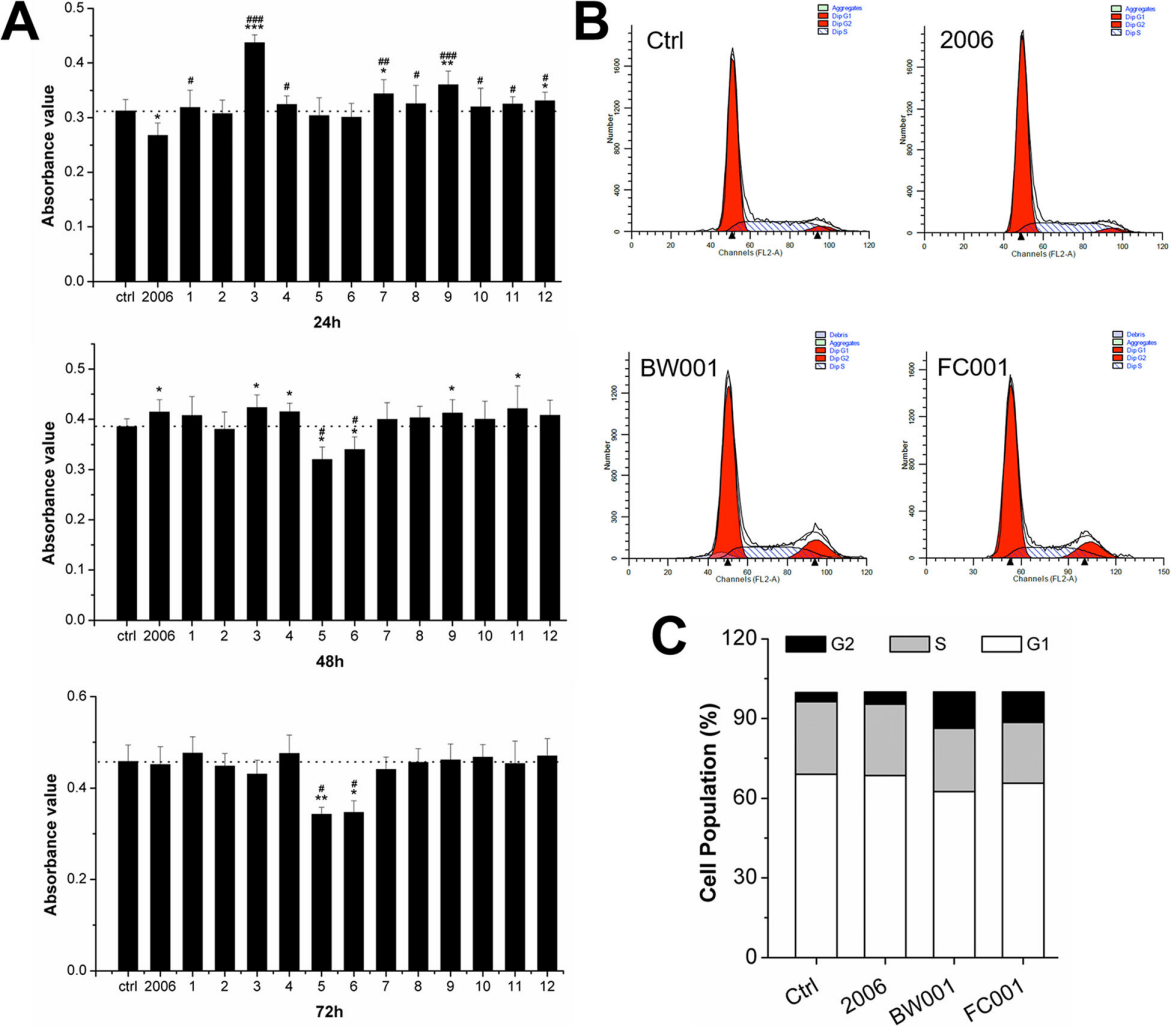
Effects of 13 CpG ODNs on cell proliferation of MCT3T cells. **a** MTT assay detected MC3T3 cell viability co-cultured with 12 ODNs, ODN 2006 and PBS for 24, 48 and 72 h. 1, FC003; 2, SAT05f; 3, SAT05d; 4, MS19; 5, BW001; 6, FC001; 7, FC002; 8, BW006; 9, YW002; 10, YW001; 11, FC004; 12, MT01. *n* = 6. *P < 0.05, **P < 0.01 and ***P < 0.001 compared with the control group and ^#^*P* < 0.05, ^##^*P* < 0.01 and ^###^*P* < 0.001 compared with the ODN 2006 group by paired *t* test. **b** Cell cycle examination by flow cytometry after co-culture for 72 h. The abscissa indicates FL2-A channels, and the ordinate indicates cell number. **c** Quantative analysis of cell population in flow cytometry. *n* = 3. Data represent the mean ± SD of three independent experiments

Bone regeneration mainly included proliferation and differentiation of osteoblasts. Therefore, we further investigated the effect of ODNs on MC3T3 cell proliferation. The MTT assay revealed that most ODNs could significantly increase cell proliferation compared with control group except for BW001 and FC001 (number 5 and 6, *P* < 0.05) at 24 h. However, these effects of ODNs on cell proliferation gradually tended to be reduced along with the treating time. At 72 h, only BW001 and FC001 showed significant declining cell viability compared with control group and positive control group (*P* < 0.05, Fig. 2a). As a result, we presented that the effect of CpG ODN on proliferation of MC3T3 cells occurred in the early stage, and gradually weakened to normal. However, it is interestingly that the inhibition effect of BW001 and FC001 on proliferation retained with extended time.

In order to investigate the underlying mechanism of inhibition of cell proliferation of BW001 and FC001, we further conducted cell cycle assay. The flow cytometry results revealed that BW001 and FC001 could decrease the cell percentage of G1 and S phase, but greatly increase the cell percentage of G2 phase, compared with both PBS and ODN 2006 groups (Fig. 2b, c). These results suggested that BW001 and FC001 might inhibit cell proliferation by decreasing cells in G1 and S phase and blocking cells in G2 phase.

### CpG ODN regulated osteogenic differentiation of MC3T3 cells

**Fig. 3:**
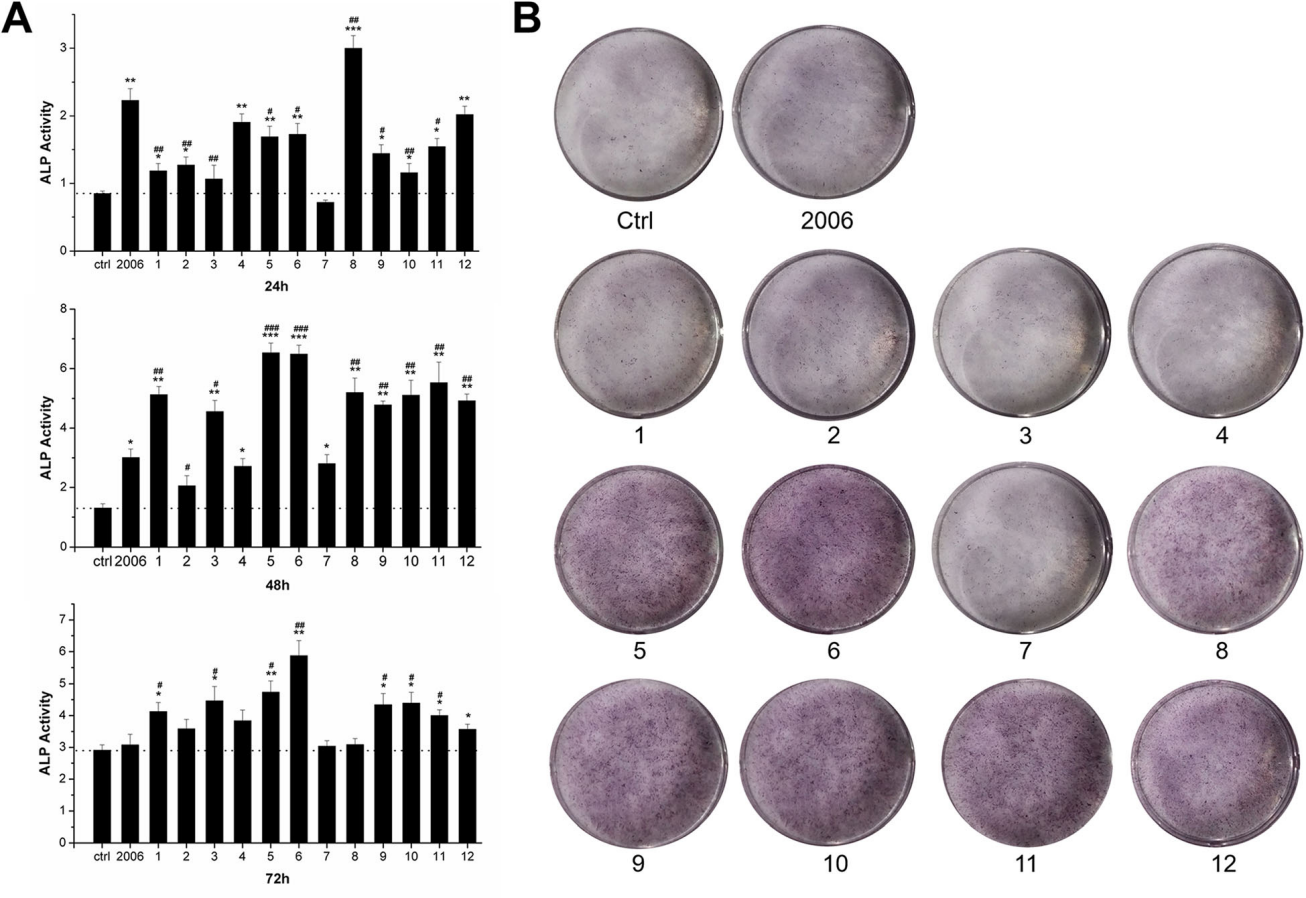
Effects of ODNs on osteogenic differentiation. **a** ALP activity assay and **b** ALP staining assay of MC3T3 cells treated by 12 ODNs for 24 h, 48 h, 72 h, and 7 days, respectively. 1, FC003; 2, SAT05f; 3, SAT05d; 4, MS19; 5, BW001; 6, FC001; 7, FC002; 8, BW006; 9, YW002; 10, YW001; 11, FC004; 12, MT01. *n* = 3. Data were expressed as mean ± SD of three independent experiments. *P < 0.05, **P < 0.01 and ***P < 0.001 compared with the control group and ^#^*P* < 0.05, ^##^*P* < 0.01 and ^###^*P* < 0.001 compared with the ODN 2006 group by paired *t* test

Subsequently, we detected the effect of ODN on MC3T3 cell differentiation. To select the effective ODNs on MC3T3 cell differentiation, we carried out ALP activity detection. As shown in Fig. 3a, except for FC002 and SAT05f, all the other ODNs could markedly improve the ALP activity of MC3T3 cells at 24 and 48 h, compared with the control group (Fig. 3a). Similar with the MTT assay, we found these effects were attenuated along with treatment time, except for BW001 and FC001, which demonstrated a significantly consistent increase over time (*P* < 0.05). The ALP staining test at 7 days showed that BW001, FC001, BW006, YW002, YW001, FC004 and MT01 kept significant promotion effect of MC3T3 cell differentiation (Fig. 3b). Therefore, we selected ODNs of BW001, FC001, BW006, YW002, YW001 and FC004 for the following experimental study.

### CpG ODNs could increase the relative expression of osteogenic differentiation genes SP7 and OCN

**Fig. 4:**
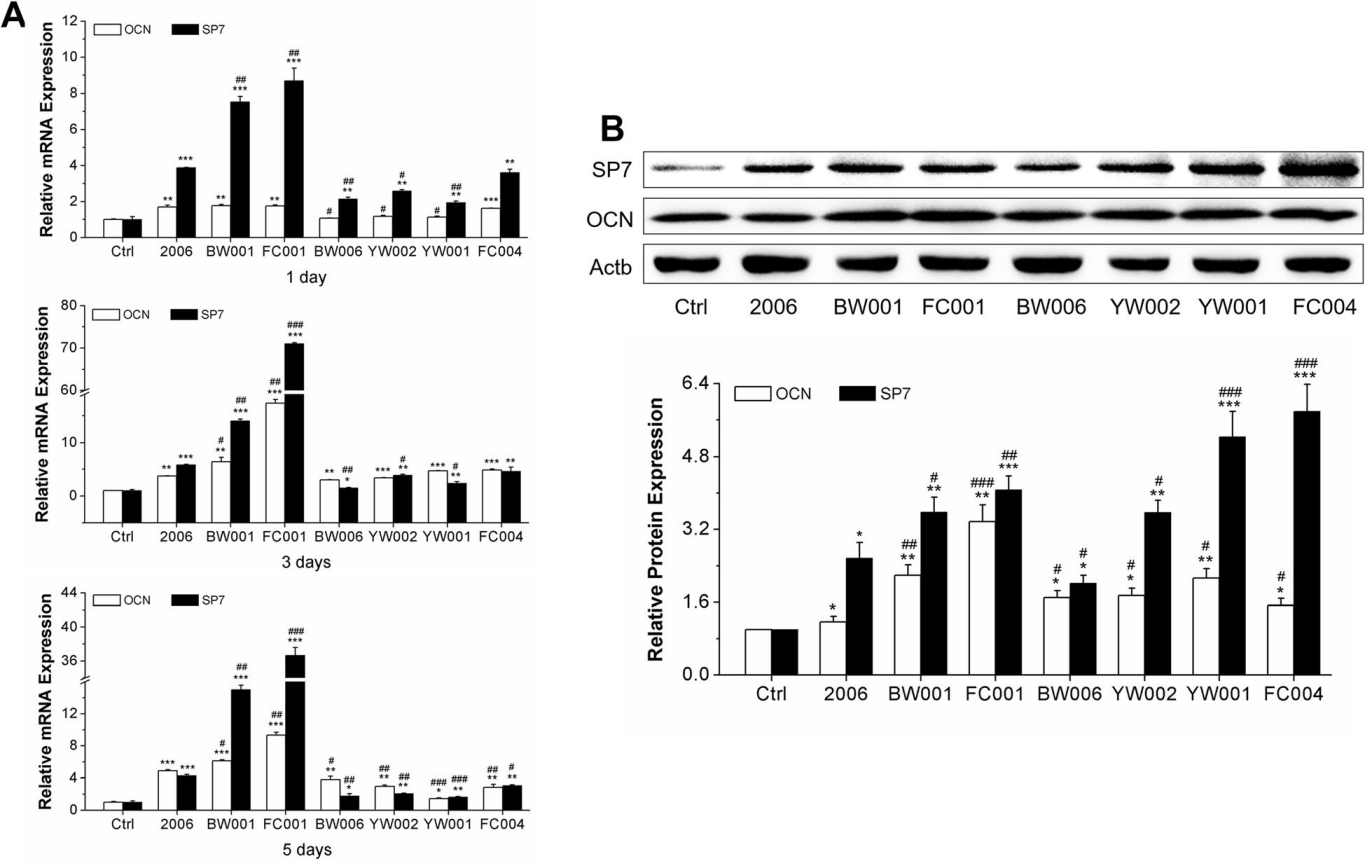
Effects of ODNs on osteogenic differentiation gene expression. **a** Quantitative real-time PCR analysis of SP7 and OCN mRNA expression of MC3T3 cells treated with selected CpG ODNs for 1 day, 3 days and 5 days. *n* = 3. **b** Western blot analysis of SP7 and OCN protein levels of MC3T3 cells treated with selected CpG ODNs for 3 days. *n* = 3. Data were expressed as mean ± SD of three independent experiments. *P < 0.05, **P < 0.01 and ***P < 0.001 compared with the control group and ^#^*P* < 0.05, ^##^*P* < 0.01 and ^###^*P* < 0.001 compared with the ODN 2006 group by paired *t* test

The relative expression levels of osteogenic differentiated genes, SP7 and OCN, were detected through qRT-PCR assay at 1 day, 3 days and 5 days. We found that the relative expression of SP7 and OCN were significantly upregulated by these CpG ODNs and the relative expression level reached to the peak on day 3 (*P* < 0.05). In addition, the upregulation of SP7 was more significant than that of OCN. Moreover, the effect of BW001 and FC001 on the increasing gene level was significantly higher than that in the ODN 2006 group, while the effect of BW006, YW002, YW001 and FC004 was weaker (Fig. 4a). Western blot results exhibited that the six selected CpG ODNs significantly promoted the protein expression of SP7 and OCN, and the promotion of SP7 was stronger than OCN, which were consistent with the results of qRT-PCR (Fig. 4b). Therefore, BW001, FC001, BW006, YW002, YW001 and FC004 improved the osteogenic differentiation of MC3T3 cells at the gene and protein levels.

### BW001 and FC001 CpG ODN decrease the protein expression of cyclin D1 and promote the expression of Runx2

**Fig. 5:**
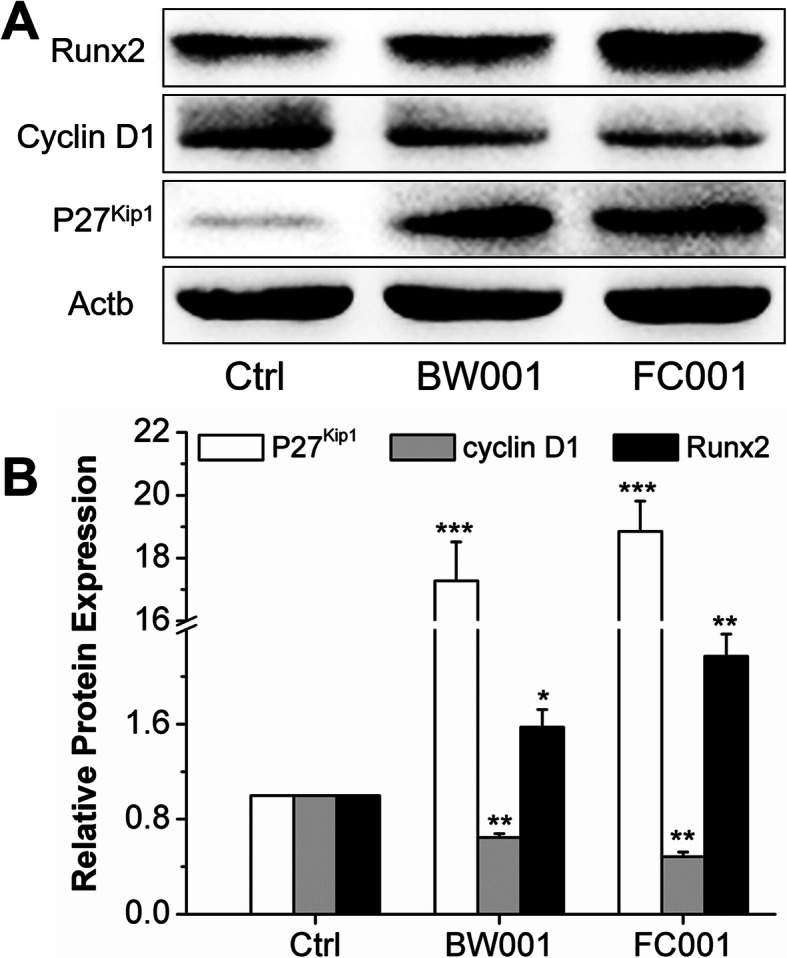
Effects of BW001 and FC001 on protein expression of the cyclin D1/Runx2 regulation network. **a** Western blot analysis of P27^Kip1^, cyclin D1 and Runx2 protein levels of MC3T3 cells treated with BW001 and FC001 for 3 days. **b** Quantative analysis of western blot analysis. Data were expressed as mean ± SD of three independent experiments. *P < 0.05 vs. the control group by paired *t* test

In previous results, BW001 and FC001 exhibited an inverse association between the proliferation and differentiation of MC3T3 cells. To explore the potential mechanism of different effect of BW001 and FC001 on MC3T3 cell proliferation and differentiation, we examined the protein expression of cyclin D1 and Runx2 under treatment of BW001 and FC001. As shown in Fig. 5, BW001 and FC001 induced significant expression of P27^Kip1^, which was an inhibitor of cyclin/CDKs (like cyclin D1, cyclin A and cyclin E). With the elevation of P27^Kip1^, protein expression of cyclin D1 was decreased while the expression of Runx2 was enhanced by BW001 and FC001 (Fig. 5).

## Discussion

In this study, 12 kinds of CpG ODNs including FC003, SAT05f, SAT05d, MS19, BW001, FC001, FC002, BW006, YW002, YW001, FC004 and MT01 were synthesized, and their roles on proliferation and differentiation of osteoclasts were investigated. Results showed that 90% CpG ODN could be uptaken by MC3T3 cells in 1 h and located in the cell cytoplasm. MTT assay demonstrated CpG ODNs could promote MC3T3 cell proliferation and differentiation in the early stage, and gradually attenuated along with the increase of treating time, except for BW001 and FC001. qRT-PCR assay demonstrated that all the 12 CpG ODNs could promote the relative expression level of osteogenic differentiated genes, SP7 and OCN. In addition, western blot analysis suggested the CpG ODNs of BW001 and FC001 could increase the protein expression of P27^Kip1^ and Runx2 and decrease the protein expression of cyclin D1.

CpG ODN is widely used in the research and treatment of immunology. However, crucial problems, such as degradability by nuclease and low uptake by cells, would limit its wide clinical application. To overcome this disadvantage, researchers employ chemical modifications of ODN. Among them, phosphorothioate of ODN achieved more stability against enzymatic degradation [16, 17]. CpG ODN is uptaken into cells by receptor-mediated endocytosis, and this process requires amount of energy and is closely related to temperature and time [18]. CpG ODN enters the endosomes via PI3K, then specially interacted by TLR9 in the endosomes. The MT01 ODN was confirmed to enter MG63 cells by endocysis in Hou’s study [19], and the uptake of MT01 ODN became to increase to peak on 12 h. However, in this study, CpG ODN reached more than 90% tranfection efficiency in 1 h. In addition, CpG ODN can also enter into cells by delivery vehicle. In the study of Zhang [20] and Zheng [21], they respectively applied graphene oxide-chitosan nanocomposites and *N*-isopropylacrylamide-modified polyethylenimine (PEN) to achieve delivery of CpG ODN into RAW264.7 cells and MG63 cells, and both abtained higher transfection. Noteworthily, in our study, the Cy5-CpG ODN mainly located in the cytoplasm, which was similar to the results of Hou and Zhang. This was in accordance with the location of TLR9 in the endolysosome [5, 20, 22]. Therefore, the phosphorothioate CpG ODN not only rapidly and efficiently entered the cells, but also located in the cytoplasm.

Previously, Shen [12] and Feng [13] reported that MT01, SAT05d, BW001, FC002, YW001 and FC004 could be able to promote the proliferation while BW001, BW006, MT01, FC001, FC002, FC004, YW001, and YW002 could stimulate the differentiation of BMSCs to osteoblasts. Our results showed that FC003, SAT05f, SAT05d, MS19, FC002, BW006, YW002, YW001, FC004 and MT01 could promote MC3T3 cell proliferation and differentiation. These results were partly in consistent with the study of Shen and Feng. Besides, we found that the effects of these ODNs on cell proliferation and differentiation were gradually attenuated along with the increase of treating time, indicating the effect of CpG ODNs on proliferation and differentiation of MC3T3 cells occurred in the early stage. This result was in line with Feng et al. who showed that ODN FC002, MT01 and YW001 significantly induced ALP activity at 24 h while there was no statistically significant difference between them and the control group.

Besides, in our study, we found that the effect of ODNs BW001 and FC001 were different with other ODNs. They showed inhibition effects on cell proliferation and differentiation, and these effects were enhanced but not attenuated along with treating time. This result is also different with the results of Shen and Feng. Cell cycle analysis suggested that BW001 and FC001 could block MC3T3 cells in G2 phase.

Noteworthily, among these CpG ODNs, BW001 and FC001 led to an inverse effect on proliferation and differentiation of MC3T3 cells. This phenomenon could also be seen in many developmental processes, like myogenesis and neural development [23, 24]. The researchers confirmed this effect might due to the consensus motif of cyclin-CDK phosphorylation locating at the serine residue of Runx2 phosphorylation site [25, 26]. In our study, western blot analysis showed that the CpG ODNs of BW001 and FC001 could increase the protein expression of P27^Kip1^ and Runx2 and decrease the protein expression of cyclin D1. P27^Kip1^ is an inhibitor of cyclin/CDKs (like cyclin D1, cyclin A and cyclin E) [27]. Runx2 was a specific transcription factor of osteogenic differentiation [28]. The active Runx2 initiated a series of osteogenic differentiated genes, such as SP7, OCN, OPN and ALP [29, 30]. Galindo et al. confirmed that the mechanism of Runx2 inducing osteoblast differentiation involves the exit from cell cycle [31] and Runx2 can be degraded by cyclin D1-Cdk4 in an ubiquitination-proteasome-dependent manner [24]. Moreover, in our data, a dramatically enhanced Runx2 and G2 phase were clearly observed. This was in consistent with Qiao [32], who demonstrated a maximal Runx2 activity existing in late G2/M phase.

## Conclusion

The phosphorothioate CpG ODN could efficiently enter the cells without vehicles and locate in the cytoplasm. The BW001 and FC001 inhibited the proliferation of MC3T3 cells by arresting cells in G2 phase. Moreover, we discovered six CpG ODNs (BW001, FC001, BW006, YW002, YW001 and FC004) that could significantly improve the differentiation of MC3T3 cells. Noteworthily, we found the underling potential mechanism of BW001 and FC001 about inverse regulation on proliferation and differentiation might attribute to cyclin proteins/Runx2 network. The selected CpG ODNs were expected to be a potential gene therapy for treating periodontitis inflammation and bone regeneration.

## Data Availability

The datasets used and/or analyzed during the current study are available from the corresponding author on reasonable request.
