# Correction: The Toll-like receptor ligand, CpG oligodeoxynucleotides, regulate proliferation and osteogenic differentiation of osteoblast

**DOI:** 10.1186/s13018-023-04295-2

**Published:** 2023-11-02

**Authors:** Wenwen Yu, Yi Zheng, Hongyan Li, Hongbing Lin, Zhen Chen, Yue Tian, Huishan Chen, Peipei Zhang, Xiaowei Xu, Yuqin Shen

**Affiliations:** 1 Department of Periodontics, School and Hospital of Stomatology, Jilin University, 1500 Qinghua Road, Changchun, 130021 Jilin China; 2 Department of Orthodontics, Tianjin Key Laboratory of Oral and Maxillofacial Function Reconstruction; Tianjin Stomatological Hospital; Hospital of Stomatology, Nankai University, 75 Dagu North Road, Tianjin, 300041 China

**Correction: Journal of Orthopaedic Surgery and Research (2020) 15:327** 10.1186/s13018-020-01844-x

Following publication of the original article [1], the authors identified an error in Fig. 3. The correct figure is given below.

**Fig. 3:**
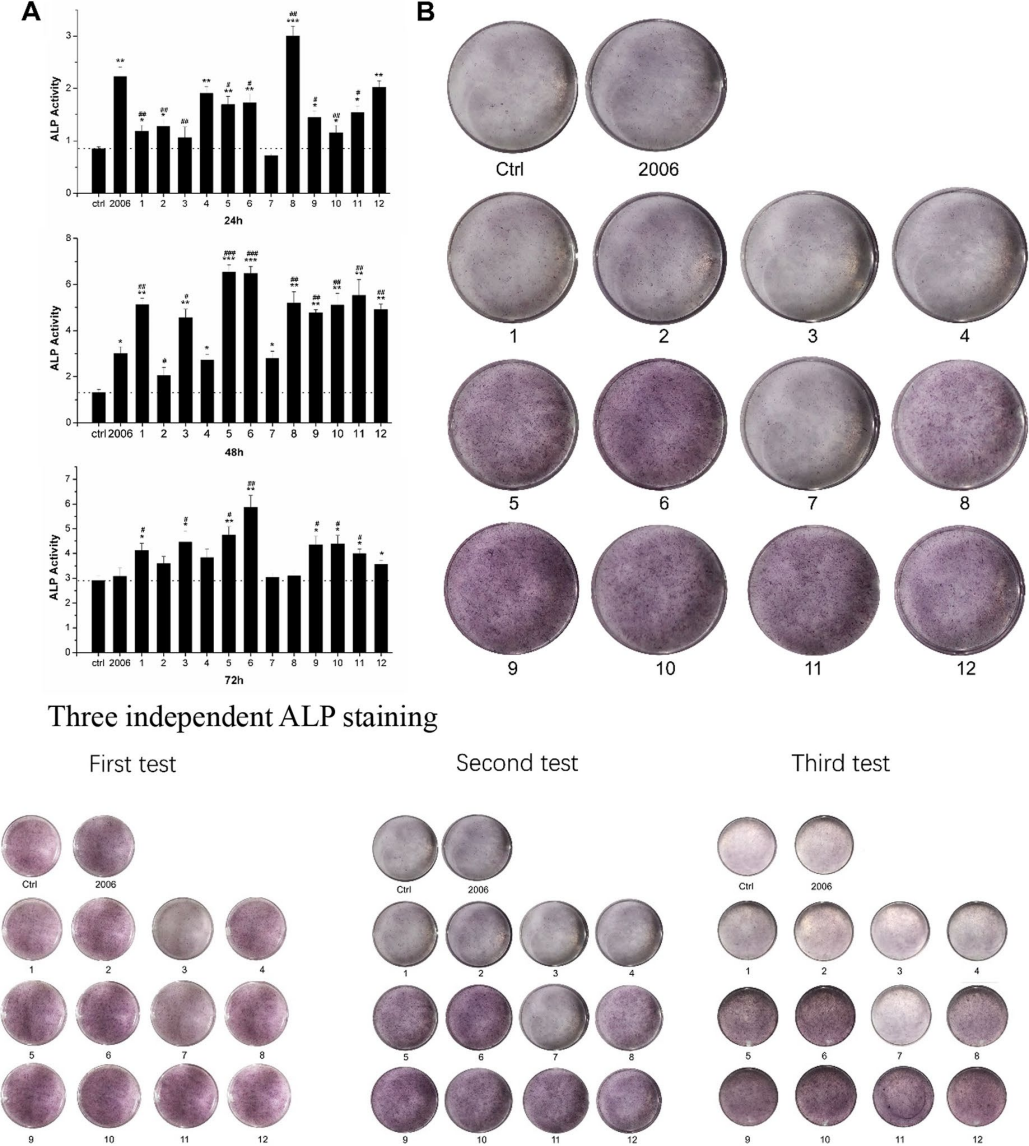
Effects of ODNs on osteogenic differentiation. **a** ALP activity assay and **b** ALP staining assay of MC3T3 cells treated by 12 ODNs for 24 h, 48 h, 72 h, and 7 days, respectively. 1, FC003; 2, SAT05f; 3, SAT05d; 4, MS19; 5, BW001; 6, FC001; 7, FC002; 8, BW006; 9, YW002; 10, YW001; 11, FC004; 12, MT01. n = 3. Data were expressed as mean ± SD of three independent experiments. *P < 0.05, **P < 0.01 and ***P < 0.001 compared with the control group and #P < 0.05, ##P < 0.01 and ###P < 0.001 compared with the ODN 2006 group by paired t test

## References

[CR1] Yu Wenwen (2020). The Toll-like receptor ligand, CpG oligodeoxynucleotides, regulate proliferation and osteogenic differentiation of osteoblast. J Orthop Surg Res.

